# Measuring the excess mortality during the COVID-19 pandemic in the Northern Territory, Australia

**DOI:** 10.1017/S0950268826101046

**Published:** 2026-02-06

**Authors:** Renu Unnikrishanan, Yuejen Zhao, Christopher Paul Burgess, Peter Gregory Markey, Ramakrishna Chondur, Jerry Chen, Vicki Krause

**Affiliations:** 1 Department of Health, Northern Territory, Australia; 2 Centre for Disease Control, Northern Territory, Australia

**Keywords:** Aboriginal population, ARIMA model, COVID-19, excess mortality, non-Aboriginal population, Northern Territory

## Abstract

This study aims to assess whether there was any excess mortality among the Aboriginal and non-Aboriginal populations in the Northern Territory (NT), Australia, during the COVID-19 pandemic. A time-series analysis using death data (1997–2023) was applied separately to the monthly and yearly death counts to develop an excess mortality surveillance model (using Auto-Regressive Integrated Moving Average (ARIMA)) for the NT population. Excess mortality was calculated by comparing expected deaths with actual deaths. In 2022, there was a statistically significant excess mortality of 193 (*p* < 0.01), compared with 82 recorded COVID-19 deaths. Excess mortality was significant for both the Aboriginal (*N* = 91) and non-Aboriginal (*N* = 102) populations in 2022. Even though some months had significant excess mortality among both Aboriginal and non-Aboriginal populations, the recorded COVID-19 deaths were not high in these months. This was associated with the peak of COVID-19 fatalities. The ARIMA model demonstrates deviations from expected deaths and helps understand the pandemic’s impact on the NT. Excess deaths occurred in 2022; however, no large spikes in most of the months suggest public health success in the NT.

## Introduction

Nationally and globally, excess mortality due to the coronavirus disease (COVID-19) was observed [1, 2]. Globally, there have been 7,070,128 COVID-19 deaths reported to the World Health Organisation (WHO) until 6 October 2024 [3], while estimated excess deaths were 14.83 million during 2020–2021 [4]. Australia reported a relatively lower number of COVID-19 deaths compared to other parts of the world [5], with a total of 20,095 COVID-19 related deaths from 2020 to August 2023 [6], and of the total deaths registered by the Australian Bureau of Statistics (ABS) until January 2024, 3.2% (21,827 of the 687,639) died from or with COVID-19. Among these, COVID-19 was the underlying cause of death for 17,276, while it contributed to a further 4,551 deaths caused primarily by other conditions (mainly chronic cardiac conditions) [7]. From August 2021 to January 2024, COVID-19 has caused 394 deaths among Aboriginal and Torres Strait Islander peoples [7]. According to the Actuaries Institute, Australia’s mortality rate was 12% higher than the expected for the full year 2022 during the global pandemic of COVID-19 [2], and excess mortality was 14.1% as per ABS for the first 8 months of 2022 [6].

With a population of 245,000 and an area of more than a million square kilometres, the Northern Territory (NT) is a geographically isolated and sparsely populated Australian jurisdiction [8]. The Howard Springs International Quarantine Facility at the Centre for National Resilience in the NT was used as Australia’s COVID-19 quarantine facility [9]. The NT population has a higher risk of COVID-19 mortality due to the higher proportion of Aboriginal population (30.8%) which has a high burden of chronic disease [8, 10, 11]. This increased disease burden, along with the geographical remoteness, increased barriers to accessing healthcare and the high mobility of the NT Aboriginal population, increases the risk of COVID-19 transmission and poor outcomes [11, 12]. It is unclear if there were excess deaths during COVID-19 in the NT.

Relying on recorded COVID-19 deaths risks underestimating the true burden of mortality due to the pandemic [13]. Not only are there issues with inaccurate case ascertainment but counting only recorded COVID-19 deaths ignores those deaths which may have arisen indirectly from the pandemic due to the impact of isolation, restricted access to routine health care and disruption of the health system [14]. Excess mortality is considered to be the most appropriate indicator [15] of the true burden, comparing actual deaths with those predicted had the pandemic not occurred [13, 16].

Our objective was to investigate whether there was excess mortality in the NT during the COVID-19 pandemic among Aboriginal and non-Aboriginal populations.

## Methods

Assessment of excess mortality follows a standard methodology to evaluate the impact of a condition on a population, and how it has changed over time, by comparing the observed number of deaths in a specific time period with the total expected deaths in that same period [16, 17]. The expected deaths for the study were modelled using pre-pandemic death data (1997 to 2019). To avoid the biased estimates of excess mortality and to remove the random mortality fluctuations which may be misinterpreted as excess mortality, the excess deaths were estimated using a time-series analysis [18]. An Auto-Regressive Integrated Moving Average (ARIMA) model was applied separately to the monthly and yearly death counts from 1997 to 2023 using Stata (SE 18.0) [19]. The choice of using counts rather than age-standardized rates was due to the lack of monthly population data and the stability of the population over the years. Using raw counts allows the model to directly analyse the observed number of deaths, and counts provide stability in analysis.

This study used the death registration data from the NT Registry of Births, Deaths and Marriages (BDM) and Cause of Death (COD) Unit Record File from Australian Coordinating Registry (ACR), to develop an excess mortality surveillance model for the NT population. Due to 2–3 year delays in reporting and coding of the ACR mortality data, all NT registered ACR COD (1997 to 2021) data were appended to BDM death registrations (January 2022 to December 2023). Recorded COVID-19 deaths were identified by using ICD-10 codes for ACR and searching text ‘COVID’ for BDM. The NT COVID-19 notifications from the NT Centre of Disease Control (CDC) were used to create the epi curve for the COVID-19 pandemic incident cases in the NT for a period March 2020 to December 2023.

This study included the NT death notifications from 1997 to 2023 based on date of death. It included all the deaths registered in the NT [20]. Excess mortality considers both direct and indirect deaths during COVID-19 [17]. We compared excess mortality with the available BDM/ACR recorded COVID-19 deaths. The COVID-19 recorded deaths in the dataset were considered to be those in which COVID-19 (ICD-10) was recorded as the underlying cause or associated causes of death on death certificates.

The monthly counts were standardized to allow for the difference in month duration. The stationarity of the data was tested [21] using visual plots, mean, variance, the autocorrelation function (ACF) and partial autocorrelation function (PACF) plots within the ARIMA modelling framework and the data made stationary by differencing. After identifying the most suitable lags for the AR and MA component, the model parameters were estimated using least squares using Stata (SE 18.0). The model with lowest degree of freedom and maximum likelihood was selected as the best ARIMA model, using Akaike information criterion (AIC) and Bayesian information criterion (BIC) [22].

The Northern Territory experiences two distinct seasons: a wet season from November to April and a dry season from May to October. The mean differences using Poisson distribution were employed to check whether significant seasonal patterns exist affecting deaths. The average numbers of deaths in two seasons were not significantly different. No statistically significant periodic spikes were observed at seasonal lags in the ACF and PACF plots, indicating an absence of detectable seasonal structure in the time series. Based on this assessment, a non-seasonal ARIMA model was chosen.

A long reference period allows time series analysis to provide a sufficient amount of historical data and accurately identify and model underlying patterns, such as trends and seasonality. This extended dataset enhances the reliability of parameter estimation, reduces the risk of overfitting, and improves the model’s ability to capture long-term predictability. Additionally, a longer reference period allows for better detection of structural changes or shifts in the data, leading to more robust and accurate forecasts.

The death data were forecasted from 2020 to 2023, by keeping 2019 as base year for projection to ascertain the expected number of deaths. Excess mortality was calculated separately for Aboriginal and non-Aboriginal populations. Univariate time series models with 95% and 99% projection intervals have been used for assessing the deaths from 2020 to 2023 and the expected death for the 4 years starting from 2020. The Ljung-Box test and residual plots were used to assess the adequacy of the ARIMA model by testing whether the residuals are white noise [21] and the forecast accuracy was evaluated using the Mean Absolute Percentage Error.

Ethics approval was granted by Human Research Ethics Committee of the NT Department of Health and Menzies School of Health Research (HREC-2023-4610).

## Results

The NT had a mean of 1,220 deaths per year during the last 4 years, from 2020 to 2023, whereas the mean number of deaths for the same period before COVID was 1,072. The total number of COVID-19 recorded deaths in the NT till the end of 2023 was 110. For the death dataset (Supplementary Figure S1 **)**, the data were made stationary after first order differencing. The model with the best fit was ARIMA (0,1,1) (Supplementary Table S1).

**Figure 1.**
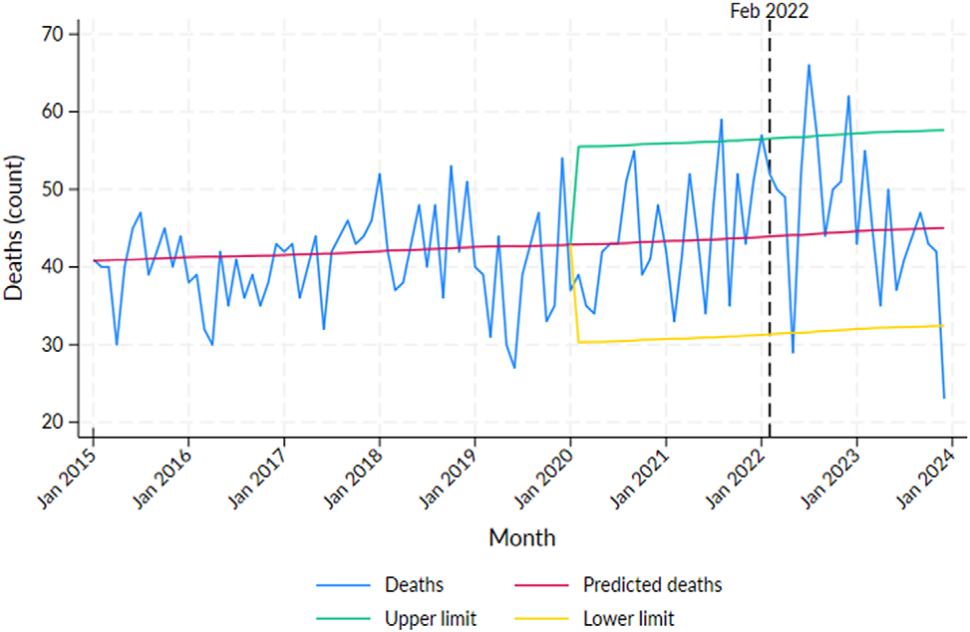
Number of deaths by month in the Northern Territory Aboriginal population from 2015 to 2023 with predicted deaths and 95% projection intervals after 2019. *Note*: Vertical line at February 2022 indicates the peak of the first wave of COVID-19 infections in the NT.

Excess mortality for the Aboriginal population was detected in 2021: August (*N* = 15, *p* < 0.05) and in 2022: January (*N* = 13, *p* < 0.05), July (*N* = 22, *p* < 0.01) and December (*N* = 18, *p* < 0.01) (Supplementary Table S2, Figure 1). The recorded COVID-19 deaths were not high in these months, with the peak of COVID-19 deaths occurring in February 2022 (*N* = 16, *p* > 0.05) (Supplementary Table S2). Months with significant declines in mortality were observed in May 2022 and December 2023.

**Figure 2.**
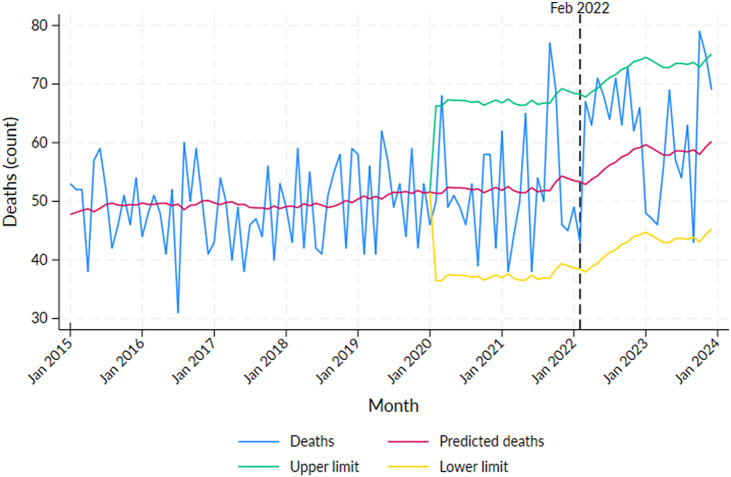
Number of deaths by month in the Northern Territory non-Aboriginal population from 2015 to 2023 with predicted deaths and 95% projection intervals after 2019. *Note*: Vertical line at February 2022 indicates the peak of the first wave of COVID-19 infections in the NT.

Excess mortality for the non-Aboriginal population was significant in 2020: March (*N* = 17, *p* < 0.05), 2021: September (*N* = 25, *p* < 0.01) and October (16, *p* < 0.05), 2022: May (*N* = 17, *p* < 0.05) and 2023: October (*N* = 21, *p* < 0.01) and November (*N* = 16, *p* < 0.05) (Supplementary Table S3, Figure 2). The recorded COVID-19 death numbers in May 2021 and November 2023 were 3 and 6, respectively, and for all other significant months, the COVID-19 deaths were 0; however, in August 2022, even though the excess mortality was not significant, the number of recorded COVID-19 deaths was 8 (Supplementary Table S3). A significant decline in mortality was observed in September 2023.

For the whole NT population, there was excess mortality detected in October 2021 (24, *p* < 0.05) and July (27, *p* < 0.01) and August (23, *p* < 0.05) in 2022 (Supplementary Table S4, Supplementary Figure S2). However, the recorded COVID-19 deaths were not high during these months except in August 2022 (14). The recorded COVID-19 deaths were highest in February 2022 (19). The mortality deficit (fewer deaths than expected) can be seen in February and June 2021.

**Figure 3.**
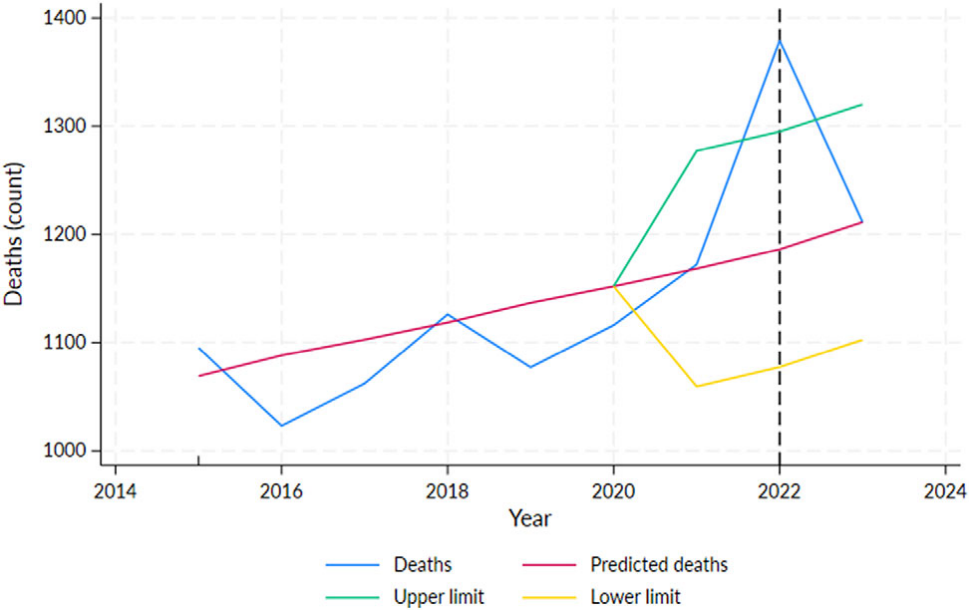
Number of deaths by year in the total Northern Territory population, 2015–2023, showing predicted deaths and the 95% projection interval after 2019. *Note*: Vertical line at February 2022 indicates the peak of the first wave of COVID-19 infections in the NT.

For the whole NT population, with yearly death data, excess mortality in 2022 was 193 (*p* < 0.01) (Figure 3), while the recorded COVID-19 deaths in that year were the highest (*N* = 82). Reduced mortality was observed in 2020 and 2023; however, it was not significant (Table 1, Supplementary Table S5). In 2021 and 2022, excess mortality was estimated at 0.3% and 16%, respectively, but was only statistically significant for 2022 (Table 1). The epi curve created using CDC COVID-19 notification data indicates that the peak time for the COVID-19 occurred in February 2022 (Supplementary Figure S3), which is consistent with the recorded COVID-19 deaths in the BDM dataset.

**Table 1.** Excess mortality for the whole population by year, Northern Territory, 2020–2023

Year	Actual deaths	Expected deaths	Excess deaths	% Excess	COVID-19 recorded deaths	COVID-19 crude death rate (per 100,000)
2020	1,116	1,151.8	−35.8	−3.1	0	0.0
2021	1,172	1,168.1	3.9	0.3	2	0.8
2022	1,379	1,186.1	192.9^a^	16.3	82	32.8
2023	1,211	1,211.3	−0.3	0.0	26	10.3

a *p* < 0.01 (based on 99% projection intervals).

Based on annual data, excess mortality for the Aboriginal (*N* = 91) and non-Aboriginal populations (*N* = 102) was significant in 2022 (*p* < 0.05), and COVID-19 deaths were also high in this year (38 and 44, respectively). In other years, observed mortality was similar to the expected deaths (Table 2).

**Table 2.** Excess mortality by year and Aboriginal status, Northern Territory, 2020–2023

	Year	Actual deaths	Expected deaths	Excess deaths	% Excess	COVID-19 recorded deaths	COVID-19 crude death rate (per 100,000)
Aboriginal	2020	507	516.8	−9.8	−1.9	0	0.0
	2021	534	522.1	11.9	2.3	1	1.3
	2022	619	528.3	90.7^a^	17.2	38	49.4
	2023	505	537.5	−32.5	−6.0	7	9.0
Non-Aboriginal	2020	609	635.0	−26.0	−4.1	0	0.0
	2021	638	646.0	−8.0	−1.2	1	0.6
	2022	760	657.8	102.2^a^	15.5	44	25.4
	2023	706	673.8	32.2	4.8	19	10.9

a *p* < 0.01 (based on 99% projection intervals).

## Discussion

The COVID-19 pandemic resulted in a significant rise in population mortality in the NT. During 2022, coinciding with the first significant waves of the pandemic in the NT, we observed excess mortality in the NT for both Aboriginal and non-Aboriginal populations. As expected in a small and dispersed population, excess mortality counts per month for the Aboriginal and non-Aboriginal population were low in 2022; however, the excess mortality counts per year were significant.

The excess mortality due to COVID-19 in Australia was relatively low compared to some other countries [23]. Australia implemented strict measures to control and slow the spread of the virus, including border closures, lockdowns, social distancing, widespread testing and contact tracing efforts, and a vaccination programme [23, 24]. Our results demonstrate that the NT experienced a significant excess mortality only in 2022. In part, this may be explained by the NT public health measures, younger population, success in the vaccination programme, widely dispersed populace and the less virulent Omicron variant that was predominant COVID-19 strain in 2022 [6, 12, 23, 25, 26]. The first case reported in the NT was on March 4, 2020 [27], and NT declared a public health emergency, implemented border restrictions and quarantine measures [28] as well as public health measures such as social distancing and stay at home measures [29]. The easing of restrictions began in May 2020, and the Territory check-in app was introduced in November [28].

COVID-19 vaccines were available to all Australians from February 2021 [25]. A staged re-opening of the international borders began by the end period of 2021, and the Omicron variant started spreading throughout the country [6]. The epi curve case numbers were insignificant until January 2022 (Supplementary Figure S3), and the first COVID-19 death happened in the NT in December 2021 leading to the introduction of indoor mask mandate and a double vaccine mandate for travellers from December 20, 2021 [28]. NT recorded its highest daily case number on February 2, 2022 (1162), and gradually eased the restriction and ended the public health emergency on June 15, 2022 [28]. All these measures might have helped in delaying and reducing the transmission and mortality rate due to COVID-19 in the NT.

The excess mortality in 2020 was negative for both Aboriginal and non-Aboriginal population in the NT. The public health measures and lockdowns might have reduced the mortality by declining both the communicable and non-communicable causes of death [23, 30, 31]. The excess mortality for the year 2021 and 2022 was 4 and 193, respectively (Table 1); however, there were only two deaths due to COVID-19 in the NT in 2021. Excess mortality in 2021 was positive for Aboriginal population and negative for non-Aboriginal population. As the resources were diverted to vaccination and public health responses in the NT, there were reduced access to care or interruption of health services in 2021, which can be the reason for the excess deaths in 2021 among Aboriginal population. The excess mortality in 2022 was significant for both Aboriginal and non-Aboriginal population, and COVID-19 deaths were also high among them in 2022. Our study showed that the NT recorded mortality at 16% above expected in 2022 (significant); however, the ABS reported a 19.4% above expected for the year 2022 in the NT [32]. The recently updated BDM data have been used for this study, and the differences may be due to the methodologies employed or surveillance systems used.

For the year 2023, we reported a slight negative (fewer than expected) or zero excess deaths, even though there were 26 COVID-19 recorded deaths. Excess mortality in 2023 was negative for Aboriginal population and positive for non-Aboriginal population. Due to the extended registration delays in the NT, there is significant uncertainty regarding the death numbers for 2023, especially for Aboriginal population. Fewer (negative) excess deaths may be due to an influence of expected deaths on previous year’s deaths. After reducing COVID-19 restrictions, it is possible that COVID-19 deaths were offset by a lower total number of deaths in 2023 as resources previously allocated to COVID-19 management are redirected towards improving the management of other leading causes of death.

With monthly data, Aboriginal and non-Aboriginal populations recorded significant excess mortality in some months during 2020 to 2023. However, the recorded COVID-19 death numbers in these months were not high. This may, in part, reflect the success of public health measures to reduce shock on the health system by ‘flattening the curve’ of transmission and hospitalizations. The excess mortality might be as a result of the indirect effect of the pandemic such as delayed care and disruption of health services. The COVID-19 death numbers and excess mortality were relatively small among Aboriginal and non-Aboriginal population. However, for the whole NT population, the excess mortality was significant in August 2022, and recorded COVID-19 death numbers were also high compared to other months.

The COVID-19 pandemic has illustrated the benefit of timely monitoring of mortality patterns and trends [15]. Excess mortality is a valuable indicator for assessing pandemic health impacts because it does not rely on case ascertainment and takes into account the indirect effects of the pandemic. The NT Aboriginal population has high burden of chronic diseases, and their life expectancy at birth is about 15 years shorter than for non-Aboriginal Territorians [10], and the COVID-19 crude death rates were higher in the Aboriginal population (Table 2). We note that a similar proportion of the excess deaths observed in the NT Aboriginal population in 2022 were attributed to COVID-19 as in the non-Aboriginal population. This may allay some concerns about low ascertainment of COVID-19 as a contributing cause of death in the NT Aboriginal population.

There were some limitations to this study. This study has not analysed the contributors to death such as age, sex and other risk factors. It could not establish whether the excess deaths were due directly to COVID-19 or due to the indirect effects of the COVID-19 pandemic, such as delayed treatment availability or due to undiagnosed health issues. The model was based on the mortality trends from 1997 to 2019, and the accuracy of the projection for the years 2020 to 2023 depends on the accuracy of the past years’ data [33]. The complexity of BDM data, especially the reporting delays with BDM (e.g., due to coronial inquest) data, may also have an influence on the mortality deficit in 2023. Another limitation of this study was the use of raw death counts rather than age-standardized rates to calculate excess mortality. While this was a pragmatic decision to ensure statistical stability in the Northern Territory’s small population, it means our analysis may not fully account for the long-term changes in the population’s size and age structure.

This study also highlights the need for BDM data to be used by the Department of Health regularly in a timely manner and linked with the existing historical mortality data, for the timely reporting of the excess mortality. Excess mortality method enables the detection of any deviation from the expected mortality rates and provides crucial information for continuous monitoring and response planning. This will serve as a tool for evaluating the impacts of the pandemic.

## Conclusion

In this study, the excess mortality during COVID-19 pandemic has been calculated for the NT Aboriginal and non-Aboriginal populations to examine potential undercounting of deaths caused by the pandemic. According to this study, there was significant excess mortality in 2022 for Aboriginal and non-Aboriginal populations during COVID-19 pandemic in the NT.

## Supporting information

10.1017/S0950268826101046.sm001 Unnikrishanan et al. supplementary material Unnikrishanan et al. supplementary material

## Data Availability

No new data were created or analysed in this study. Data sharing is not applicable to this article.

## Supplementary material

The supplementary material for this article can be found at http://doi.org/10.1017/S0950268826101046.
