# Supplementary material for: Measuring the excess mortality during the COVID-19 pandemic in the Northern Territory, Australia
Source: Epidemiol Infect. 2026 Feb 6;154:e26. doi: 10.1017/S0950268826101046 (PMC12951332; doi:10.1017/S0950268826101046)
Supplement: Unnikrishanan et al. supplementary material [file S0950268826101046sup001.docx]

**Supplementary material**

**Table S1: Comparisons of tested models (Model selection) for monthly data for the total population**

| **Model** | **df** | **LL** | **AIC** | **BIC** |
| --- | --- | --- | --- | --- |
| ARIMA(0,0,0) | 2 | -1349.7 | 2703.4 | 2710.9 |
| ARIMA(0,0,1) | 3 | -1311.0 | 2628.1 | 2639.4 |
| ARIMA(1,0,0) | 3 | -1277.6 | 2561.2 | 2572.5 |
| ARIMA(1,0,1) | 4 | -1221.4 | 2450.7 | 2465.8 |
| ARIMA(0,1,0) | 2 | -1309.1 | 2622.2 | 2629.8 |
| ARIMA(0,1,1) | 3 | -1214.8 | 2435.6 | 2446.9 |
| ARIMA(1,1,0) | 3 | -1260.3 | 2526.5 | 2537.8 |
| ARIMA(1,1,1) | 4 | -1214.7 | 2437.3 | 2452.4 |

Note: df - degree of freedom, LL - log likelihood, AIC - Akaike Information Criterion and BIC - Bayesian Information Criterion

**Figure S1: NT Death data trend**

**Table S2: Excess mortality among Aboriginal population by months, Northern Territory, 2020-23**

| Month/Year | Actual deaths | Expected deaths | Excess deaths | COVID-19 recorded deaths |
| --- | --- | --- | --- | --- |
| Jan-20 | 37 | 42.9 | -5.9 | 0 |
| Feb-20 | 39 | 42.9 | -3.9 | 0 |
| Mar-20 | 35 | 43.0 | -8.0 | 0 |
| Apr-20 | 34 | 43.0 | -9.0 | 0 |
| May-20 | 42 | 43.0 | -1.0 | 0 |
| Jun-20 | 43 | 43.0 | 0.0 | 0 |
| Jul-20 | 43 | 43.0 | 0.0 | 0 |
| Aug-20 | 51 | 43.1 | 7.9 | 0 |
| Sep-20 | 55 | 43.2 | 11.8 | 0 |
| Oct-20 | 39 | 43.2 | -4.2 | 0 |
| Nov-20 | 41 | 43.3 | -2.3 | 0 |
| Dec-20 | 48 | 43.3 | 4.7 | 0 |
| Jan-21 | 42 | 43.4 | -1.4 | 0 |
| Feb-21 | 33 | 43.4 | -10.4 | 0 |
| Mar-21 | 41 | 43.4 | -2.4 | 0 |
| Apr-21 | 52 | 43.4 | 8.6 | 0 |
| May-21 | 44 | 43.5 | 0.5 | 0 |
| Jun-21 | 34 | 43.5 | -9.5 | 0 |
| Jul-21 | 48 | 43.5 | 4.5 | 0 |
| Aug-21 | 59 | 43.6 | 15.4* | 0 |
| Sep-21 | 35 | 43.7 | -8.7 | 0 |
| Oct-21 | 52 | 43.7 | 8.3 | 0 |
| Nov-21 | 43 | 43.8 | -0.8 | 0 |
| Dec-21 | 51 | 43.8 | 7.2 | 1 |
| Jan-22 | 57 | 43.9 | 13.1* | 1 |
| Feb-22 | 52 | 44.0 | 8.0 | 16 |
| Mar-22 | 50 | 44.0 | 6.0 | 4 |
| Apr-22 | 49 | 44.1 | 4.9 | 3 |
| May-22 | 29 | 44.1 | -15.1* | 0 |
| Jun-22 | 52 | 44.1 | 7.9 | 0 |
| Jul-22 | 66 | 44.2 | 21.8** | 1 |
| Aug-22 | 57 | 44.3 | 12.7 | 6 |
| Sep-22 | 44 | 44.4 | -0.4 | 1 |
| Oct-22 | 50 | 44.4 | 5.6 | 0 |
| Nov-22 | 51 | 44.5 | 6.5 | 4 |
| Dec-22 | 62 | 44.5 | 17.5** | 2 |
| Jan-23 | 43 | 44.6 | -1.6 | 3 |
| Feb-23 | 55 | 44.7 | 10.3 | 2 |
| Mar-23 | 45 | 44.7 | 0.3 | 0 |
| Apr-23 | 35 | 44.8 | -9.8 | 1 |
| May-23 | 50 | 44.8 | 5.2 | 1 |
| Jun-23 | 37 | 44.9 | -7.9 | 0 |
| Jul-23 | 41 | 44.9 | -3.9 | 0 |
| Aug-23 | 44 | 44.9 | -0.9 | 0 |
| Sep-23 | 47 | 44.9 | 2.1 | 0 |
| Oct-23 | 43 | 45.0 | -2.0 | 0 |
| Nov-23 | 42 | 45.0 | -3.0 | 0 |
| Dec-23 | 23 | 45.0 | -22.0** | 0 |

* *p*<0.05, ***p*<0.01 (based on 95% and 99% projection intervals)

**Table S3: Excess mortality among non-Aboriginal population by months, Northern Territory, 2020-2023**

| Month/Year | Actual deaths | Expected deaths | Excess deaths | COVID-19 recorded deaths |
| --- | --- | --- | --- | --- |
| Jan-20 | 46 | 51.6 | -5.6 | 0 |
| Feb-20 | 50 | 51.4 | -1.4 | 0 |
| Mar-20 | 68 | 51.4 | 16.6* | 0 |
| Apr-20 | 49 | 52.4 | -3.4 | 0 |
| May-20 | 51 | 52.3 | -1.3 | 0 |
| Jun-20 | 49 | 52.3 | -3.3 | 0 |
| Jul-20 | 46 | 52.2 | -6.2 | 0 |
| Aug-20 | 53 | 52.0 | 1.0 | 0 |
| Sep-20 | 39 | 52.1 | -13.1 | 0 |
| Oct-20 | 58 | 51.5 | 6.5 | 0 |
| Nov-20 | 58 | 51.9 | 6.1 | 0 |
| Dec-20 | 42 | 52.4 | -10.4 | 0 |
| Jan-21 | 62 | 51.9 | 10.1 | 0 |
| Feb-21 | 38 | 52.5 | -14.5 | 0 |
| Mar-21 | 44 | 51.8 | -7.8 | 0 |
| Apr-21 | 50 | 51.5 | -1.5 | 0 |
| May-21 | 65 | 51.5 | 13.5 | 0 |
| Jun-21 | 38 | 52.3 | -14.3 | 0 |
| Jul-21 | 54 | 51.6 | 2.4 | 0 |
| Aug-21 | 50 | 51.8 | -1.8 | 0 |
| Sep-21 | 77 | 51.8 | 25.2** | 0 |
| Oct-21 | 69 | 53.3 | 15.7* | 0 |
| Nov-21 | 46 | 54.3 | -8.3 | 0 |
| Dec-21 | 45 | 53.9 | -8.9 | 1 |
| Jan-22 | 49 | 53.5 | -4.5 | 1 |
| Feb-22 | 43 | 53.4 | -10.4 | 3 |
| Mar-22 | 67 | 52.9 | 14.1 | 6 |
| Apr-22 | 63 | 53.7 | 9.3 | 4 |
| May-22 | 71 | 54.4 | 16.6* | 3 |
| Jun-22 | 68 | 55.4 | 12.6 | 1 |
| Jul-22 | 64 | 56.2 | 7.8 | 4 |
| Aug-22 | 71 | 56.7 | 14.3 | 8 |
| Sep-22 | 63 | 57.6 | 5.4 | 2 |
| Oct-22 | 73 | 58.0 | 15.0 | 4 |
| Nov-22 | 62 | 58.9 | 3.1 | 5 |
| Dec-22 | 66 | 59.2 | 6.8 | 3 |
| Jan-23 | 48 | 59.6 | -11.6 | 0 |
| Feb-23 | 47 | 59.1 | -12.1 | 0 |
| Mar-23 | 46 | 58.5 | -12.5 | 0 |
| Apr-23 | 56 | 57.9 | -1.9 | 2 |
| May-23 | 69 | 57.9 | 11.1 | 5 |
| Jun-23 | 57 | 58.6 | -1.6 | 2 |
| Jul-23 | 54 | 58.6 | -4.6 | 0 |
| Aug-23 | 63 | 58.4 | 4.6 | 0 |
| Sep-23 | 43 | 58.8 | -15.8* | 2 |
| Oct-23 | 79 | 58.0 | 21.0** | 0 |
| Nov-23 | 75 | 59.3 | 15.7* | 6 |
| Dec-23 | 69 | 60.2 | 8.8 | 2 |

* *p*<0.05, ***p*<0.01 (based on 95% and 99% projection intervals)

**Table S4: Excess Mortality for the whole population by month, Northern Territory, 2020-2023**

| Month/Year | Actual deaths | Expected deaths | Excess deaths | COVID-19 recorded deaths |
| --- | --- | --- | --- | --- |
| Jan-20 | 83 | 92.8 | -9.8 | 0 |
| Feb-20 | 89 | 92.2 | -3.2 | 0 |
| Mar-20 | 103 | 92.1 | 10.9 | 0 |
| Apr-20 | 83 | 93.1 | -10.1 | 0 |
| May-20 | 93 | 92.4 | 0.6 | 0 |
| Jun-20 | 92 | 92.6 | -0.6 | 0 |
| Jul-20 | 89 | 92.7 | -3.7 | 0 |
| Aug-20 | 104 | 92.5 | 11.5 | 0 |
| Sep-20 | 94 | 93.6 | 0.4 | 0 |
| Oct-20 | 97 | 93.7 | 3.3 | 0 |
| Nov-20 | 99 | 94.1 | 4.9 | 0 |
| Dec-20 | 90 | 94.6 | -4.6 | 0 |
| Jan-21 | 104 | 94.4 | 9.6 | 0 |
| Feb-21 | 71 | 95.3 | -24.3* | 0 |
| Mar-21 | 85 | 93.5 | -8.5 | 0 |
| Apr-21 | 102 | 93.0 | 9.0 | 0 |
| May-21 | 109 | 93.8 | 15.2 | 0 |
| Jun-21 | 72 | 95.1 | -23.1* | 0 |
| Jul-21 | 102 | 93.4 | 8.6 | 0 |
| Aug-21 | 109 | 94.3 | 14.7 | 0 |
| Sep-21 | 112 | 95.6 | 16.4 | 0 |
| Oct-21 | 121 | 97.0 | 24.0* | 0 |
| Nov-21 | 89 | 99.0 | -10.0 | 0 |
| Dec-21 | 96 | 98.4 | -2.4 | 2 |
| Jan-22 | 106 | 98.3 | 7.7 | 2 |
| Feb-22 | 95 | 99.0 | -4.0 | 19 |
| Mar-22 | 117 | 98.9 | 18.1 | 10 |
| Apr-22 | 112 | 100.4 | 11.6 | 7 |
| May-22 | 100 | 101.5 | -1.5 | 3 |
| Jun-22 | 120 | 101.5 | 18.5 | 1 |
| Jul-22 | 130 | 103.1 | 26.9** | 5 |
| Aug-22 | 128 | 105.3 | 22.7* | 14 |
| Sep-22 | 107 | 107.2 | -0.2 | 3 |
| Oct-22 | 123 | 107.4 | 15.6 | 4 |
| Nov-22 | 113 | 108.7 | 4.3 | 9 |
| Dec-22 | 128 | 109.2 | 18.8 | 5 |
| Jan-23 | 91 | 110.8 | -19.8 | 3 |
| Feb-23 | 102 | 109.4 | -7.4 | 2 |
| Mar-23 | 91 | 108.9 | -17.9 | 0 |
| Apr-23 | 91 | 107.7 | -16.7 | 3 |
| May-23 | 119 | 106.5 | 12.5 | 6 |
| Jun-23 | 94 | 107.6 | -13.6 | 2 |
| Jul-23 | 95 | 106.7 | -11.7 | 0 |
| Aug-23 | 107 | 105.9 | 1.1 | 0 |
| Sep-23 | 90 | 106.1 | -16.1 | 2 |
| Oct-23 | 122 | 105.0 | 17.0 | 0 |
| Nov-23 | 117 | 106.4 | 10.6 | 6 |
| Dec-23 | 92 | 107.4 | -15.4 | 2 |

* *p*<0.05, ***p*<0.01 (based on 95% and 99% projection intervals)

**Figure S2: Number of deaths by month for the whole Northern Territory population from 2015 to 2023 with predicted deaths and 95% projection intervals after 2019**

Note: vertical line at February 2022 indicates the peak of the first wave of COVID-19 infections in the NT

**Figure S3: Epidemiological Curve of COVID-19 daily incident cases in the Northern Territory: March 2020 - January 2024**

Note: vertical line at February 2022 indicates the peak of the first wave of COVID-19 infections in the NT

**Figure S4: Number of deaths by month for the whole Northern Territory population from 1997 to 2023 with predicted deaths and 95% projection intervals after 2019**

**Table S5:** Excess mortality for the whole population by year with prediction interval, Northern Territory, 2020-2023

| Year | Actual deaths | Expected deaths | Excess deaths (prediction Interval) | % Excess  (prediction Interval) | COVID-19 recorded deaths | COVID-19 crude death rate (per 100000) |
| --- | --- | --- | --- | --- | --- | --- |
| 2020 | 1116 | 1151.8 | -35.8 (-144.9, 73.3) | -3.1 (-11.5, 7.0) | 0 | 0 |
| 2021 | 1172 | 1168.1 | 3.9 (-105.1, 112.8) | 0.3 (-8.2, 10.7) | 2 | 0.8 |
| 2022 | 1379 | 1186.1 | 192.9** (84.1, 301.8) | 16.3 (6.5, 28.0) | 82 | 32.8 |
| 2023 | 1211 | 1211.3 | -0.3 (-109.1, 108.1) | 0 (-8.3, 9.8) | 26 | 10.3 |

***p*<0.01 (based on 99% projection intervals)
